# The impact of admission diagnosis on gastric emptying in critically ill patients

**DOI:** 10.1186/cc5685

**Published:** 2007-02-08

**Authors:** Nam Q Nguyen, Mei P Ng, Marianne Chapman, Robert J Fraser, Richard H Holloway

**Affiliations:** 1 Department of Gastroenterology, Royal Adelaide Hospital, North Terrace, Adelaide, 5000, Australia; 2 Department of Medicine, University of Adelaide, Frome Road, Adelaide, 5000, Australia; 3 Department of Intensive Care, Royal Adelaide Hospital, North Terrace, Adelaide, 5000, Australia

## Abstract

**Introduction:**

Disturbed gastric emptying (GE) occurs commonly in critically ill patients. Admission diagnoses are believed to influence the incidence of delayed GE and subsequent feed intolerance. Although patients with burns and head injury are considered to be at greater risk, the true incidence has not been determined by examination of patient groups of sufficient number. This study aimed to evaluate the impact of admission diagnosis on GE in critically ill patients.

**Methods:**

A retrospective review of patient demographics, diagnosis, intensive care unit (ICU) admission details, GE, and enteral feeding was performed on an unselected cohort of 132 mechanically ventilated patients (94 males, 38 females; age 54 ± 1.2 years; admission Acute Physiology and Chronic Health Evaluation II [APACHE II] score of 22 ± 1) who had undergone GE assessment by ^13^C-octanoic acid breath test. Delayed GE was defined as GE coefficient (GEC) of less than 3.20 and/or gastric half-emptying time (t50) of more than 140 minutes.

**Results:**

Overall, 60% of the patients had delayed GE and a mean GEC of 2.9 ± 0.1 and t50 of 163 ± 7 minutes. On univariate analysis, GE correlated significantly with older age, higher admission APACHE II scores, longer length of stay in ICU prior to GE measurement, higher respiratory rate, higher FiO_2 _(fraction of inspired oxygen), and higher serum creatinine. After these factors were controlled for, there was a modest relationship between admission diagnosis and GE (*r *= 0.48; *P *= 0.02). The highest occurrence of delayed GE was observed in patients with head injuries, burns, multi-system trauma, and sepsis. Delayed GE was least common in patients with myocardial injury and non-gastrointestinal post-operative respiratory failure. Patients with delayed GE received fewer feeds and stayed longer in ICU and hospital compared to those with normal GE.

**Conclusion:**

Admission diagnosis has a modest impact on GE in critically ill patients, even after controlling for factors such as age, illness severity, and medication, which are known to influence this function.

## Introduction

Enteral feeding via the nasogastric route is the preferred method of nutrition in critically ill patients [1-3]. Continuous infusion of liquid nutrient into the stomach is convenient, minimally invasive, and cost-effective. However, impaired tolerance to gastric feeding is common [4,5] and leads to patient discomfort, an increased risk of pulmonary aspiration, and delay in achieving nutritional goals with the need for prokinetic agents, post-pyloric feeding, or parenteral nutrition [3-5]. These complications of feed intolerance can adversely affect patient morbidity and mortality [6-9].

Feed intolerance is an indirect marker of disturbed gastric motility and delayed gastric emptying (GE) [3-5,9]. Slow GE in critically ill patients results from disturbed motor function of both proximal and distal stomach [10-12], but the precise mechanisms underlying these disturbances remain unclear. Several factors related to critical illness have been reported to be associated with gastric dysmotility and feed intolerance, including hyperglycaemia, the nature of acute illness, mechanical ventilation, sedatives, cytokine release, and splanchnic hypoperfusion due to shock and sepsis [2-5,13]. Critically ill patients admitted with traumatic brain injury and burns are believed to be at the highest risk of delayed GE and feed intolerance [3,4,14-18] (prevalence of up to 80%). However, data on the incidence of delayed GE in patients with other diagnoses such as sepsis and multi-trauma are limited and the techniques used to measure GE in some studies suboptimal. The phenol red test and gastric residual volume [14,18] have not been validated in humans. The paracetamol absorption test [16,17], used in many studies for indirect assessment of GE, may be less sensitive than other approaches because the first-pass metabolism of paracetamol is frequently altered in critically ill patients as a consequence of liver dysfunction or drug interactions [19]. In addition, this technique has not been validated against scintigraphy for measurement of GE in critically ill patients. In contrast, ^13^C-octanoic acid breath test [20,21] has also been compared with the 'gold standard' technique, gastric scintigraphy, and a strong correlation between the two techniques has been demonstrated in critically ill patients [22]. The aims of this study were to examine (a) the impact of admission diagnosis on delayed GE and (b) factors associated with delayed GE in critical illness by means of a validated and reliable technique for the measurement of GE, the ^13^C-octanoic acid breath test [20,21].

## Materials and methods

### Subjects

Data from an unselected cohort of critically ill patients, who were admitted to a mixed surgical and medical intensive care unit (ICU) from 1999 to 2005, were pooled from six previous clinical studies that involved measurement of GE by ^13^C-octanoic acid breath tests. Four of the studies examined the impact of critical illness on GE and motility in an unselected cohort of critically ill patients. The specific aims of each study were as follows: study 1, to evaluate the prevalence of delayed GE function (*n *= 45; data of 30 patients from this group were published in a study by Ritz and colleagues [21]); study 2, to examine the relationship between GE and antro-pyloro-duodenal motility (*n *= 18; published in a study by Chapman and colleagues [12]); study 3, to assess the impact of morphine and midazolam versus propofol on GE (*n *= 14; unpublished data); and study 4, to examine the impact of early versus delayed feeding on GE in critically ill patients (*n *= 24; unpublished data). The other two studies were randomised, placebo-controlled trials that assessed the effects of a single dose of cephalosporin (50 mg; *n *= 14; published in a study by Chapman and colleagues [23]) and erythromycin (200 mg; *n *= 30; data of 20 patients from this group were published in a study by Chapman and colleagues [24]) on GE. From the latter two trials, only the results of GE assessment during administration of placebo therapy (20 ml of normal saline) were included in this audit.

Although the outcome measures in each trial varied, the inclusion and exclusion criteria as well as GE technique (^13^C-octanoic acid breath test) were identical. Patients who participated in the trials were critically ill, required mechanical ventilation, and were able to receive nasogastric feeding. The exclusion criteria were recent major abdominal surgery, any contraindication to the passage of an enteral tube, previous gastric, oesophageal, or intestinal surgery, suspected bowel obstruction or perforation, and use of prokinetic therapy within the previous 24 hours. In all critically ill patients, written informed consent was obtained from the next of kin prior to the study. Both the audit and the assessment of GE were approved by the Research Ethics Committee of the Royal Adelaide Hospital (Adelaide, Australia).

### Data collection and analysis

All relevant details related to the patients and the ICU admission were obtained from case notes and intensive care charts. Patient demographics, admission diagnosis, Acute Physiology and Chronic Health Evaluation II (APACHE II) score, medication (prior and during admission), past medical history, blood glucose concentration, blood biochemistry, ventilation details, length of stay prior to the assessment of GE, and length of hospital stay were recorded. The APACHE II score was determined in all patients by means of a previously published method [25]. The mean rate of nasogastric feed delivery, before and after the assessment of GE, was also documented.

The patients were categorised into six major admission diagnoses: (a) intra-cerebral injury, (b) burns, (c) multi-system trauma resulting from either motor vehicle accident or fall, (d) sepsis, (e) respiratory failure after a non-abdominal surgery requiring ventilation in ICU, and (f) ischaemic myocardial injury with cardiogenic shock and significant pulmonary oedema. The 'intra-cerebral injury' category encompassed open or closed head injury related to mechanical trauma, sub-dural, sub-arachnoid, or intra-cerebral haemorrhage, and major ischaemic cerebral events. A patient was deemed to have 'sepsis' if there were clinical signs of systemic inflammatory response syndrome (SIRS) with documented evidence of infection on bacteriological assessment [26]. SIRS was recognised by the presence of two or more of the following: temperature above 38°C or below 36°C, heart rate above 90 beats per minute, respiratory rate above 20 breaths per minute or PaCO_2 _(arterial partial pressure of carbon dioxide) below 32 mm Hg, white cell count greater than 12,000 cells per cubic millimetre or less than 4,000 cells per cubic millimetre, or a blood picture showing a proportion of immature white cells of more than 10% [26].

### Technique of GE assessment: ^13^C-octanoic acid breath test

In all patients, the breath test was performed using an identical standardised technique [20-22]. Studies were performed in the morning with subjects supine in the 30-degree head-up position. In patients, enteral feeding was ceased four hours before the study. After verifying the correct position of the nasogastric tube (12-French Flexiflo [Ross Products, a division of Abbott Laboratories, Abbott Park, IL, USA] or 14-French Levin tube [Maersk Medical, Lynge, Denmark]) by routine clinical radiography or air insufflation and measurement of the gastric fluid pH, all gastric contents were aspirated and discarded. In both patients and healthy subjects, 100 μl of ^13^C-octanoate (100 mg/ml; Cambridge Isotope Laboratories, Inc., Andover, MA, USA) was added to 100 ml of Ensure™ (Abbott Australasia Pty. Ltd., Botany, NSW, Australia), a liquid meal that contains 106 kcal/100 ml. The labelled Ensure™ was shaken for one minute to distribute the marker in the meal before it was infused into the stomach over five minutes. Breath samples were obtained before meal instillation, every 5 minutes for 1 hour and every 15 minutes for a further 3 hours.

#### Collection of breath samples

In patients, end-expiratory breath samples were collected from the ventilation tube by means of a T-adapter (Datex-Engstrom, a division of Instrumentarium Corporation Helsinki, Finland) and holder for vacutainers (blood needle holder; Reko PTY Ltd, Lisarow, NSW, Australia), containing a needle (VenoJect^®^; Terumo Corporation, Tokyo, Japan). Previous data suggested that equilibration of CO_2 _concentration between the ventilation tube and evacuated 10-ml tubes (Exetainer^®^; Labco Limited, High Wycombe, Buckinghamshire, UK) took a fraction of a second and was a reliable technique of breath sampling [21]. To avoid sampling other than end-expiratory air, samples were timed to the end-expiratory phase by observation of the patients and the time-flow curve on the ventilation monitor. In healthy subjects, end-expiratory breath samples were collected by asking them to fully breathe out into sample tubes through a straw.

#### Analysis of breath samples and GE

The concentration of CO_2 _and the percentage of ^13^CO_2 _were measured in each sample by means of an isotope ratio mass spectrometer (ABCA model 20\20; Europa Scientific, Crewe, Cheshire, UK). Samples containing less than 1% CO_2 _were regarded as being non-end-expiratory and were excluded from further analysis. The ^13^CO_2 _concentration over time was plotted and the resultant curves were used to calculate a GE coefficient (GEC), a global index for the GE rate, which accounts for the rates of both appearance and disappearance of the label in breath [20]. In addition, the gastric half-emptying time (t50) was calculated [21]. Delayed GE was defined as t50 of more than 140 minutes and/or GEC of less than 3.2 [21].

### Statistical analysis

Data are expressed as mean ± standard error of mean. Differences in demographic data and GE variables between the patients groups were compared using χ^2 ^test with Yates correction (for categorical data) and Student *t *test (for continuous data). Risk factors associated with delayed GE, such as APACHE II score, age, serum creatinine, length of ICU stay prior to the breath testing, ventilation parameters, and blood glucose concentration, were assessed using Pearson's linear regression analysis. The linear relationship between these risk factors and GE variables were confirmed on histogram and Q-Q plot. After these risk factors were controlled for, the influence of admission diagnosis on GE variables was assessed using linear and hierarchical regression model analyses. Relative risk of delayed GE, as compared to that of patients with cardiac injury, was also calculated with 95% confidence interval. The statistical software used in this study was SAS/STAT version 9.1 (SAS Institute Inc., Cary, NC, USA). A *P *value of less than 0.05 was considered as statistically significant in all analyses.

## Results

A total of 145 patients had completed breath test data available. Thirteen patients were excluded from the analysis because their case notes and/or ICU charts were unable to be retrieved. Thus, the final analysis was performed on 132 critically ill patients. Overall patient characteristics and details related to their ICU admission are summarised in Table 1. The three most common admission diagnoses were sepsis (*n *= 44), head injury (*n *= 30), and multi-system trauma (*n *= 29). Seven of 29 multi-system trauma patients also sustained head injury. Within 24 hours of GE measurement, all but 4 patients were sedated using morphine and/or midazolam alone (*n *= 48), propofol alone (*n *= 18), or a combination of these drugs (*n *= 62).

**Table 1:** Demographics of studied subjects

	Critically ill patients (*n *= 132)
Age (years)	54.4 ± 1.5
Gender (male/female)	95:37
Body mass index (kg/m^2^)	27.4 ± 0.6
Days in ICU prior to study	8.0 ± 0.6
APACHE II score	
Admission	23.9 ± 0.5
Study day	17.6 ± 0.6
Enteral feeding rate (ml/hour)	
Prior to breath testing	51.1 ± 2.9
After breath testing	56.6 ± 2.8
Diagnosis, % (*n*)	
Sepsis	33% (44)
Head injury^a^	23% (30)
Multi-trauma^a^	22% (29)
Burns	7% (9)
Non-GI post-operative respiratory compromise	9% (12)
Cardiac injury (ischaemia and failure)	11% (15)
Blood glucose level (mmol/l)	
Admission	9.7 ± 0.9
Study day	8.0 ± 0.3
Biochemistry	
Albumin (g/l)	23.6 ± 0.5
Bilirubin (μmol/l)	19.5 ± 2.5
White cell count (× 10^9^/l)	12.6 ± 0.5
Serum creatinine (mmol/l)	0.134 ± 0.01
Medications, % (*n*)	
Opioid ± benzodiazepine	83% (110)
Propofol	60% (80)
Inotropes	69% (91)
Mechanical ventilation	
SIMV/pressure support ventilation^b ^(*n*)	74:58
Fraction of inspired oxygen	0.5 ± 0.01
Positive end-expiratory pressure (cm H_2_O)	6.5 ± 0.3
Peak inspiratory pressure (cm H_2_O)	24.5 ± 0.8

^a^Seven patients had head injury due to multi-trauma. ^b^Pressure support ventilation self-triggered mode. APACHE II, Acute Physiology and Chronic Health Evaluation II; GI, gastrointestinal; ICU, intensive care unit; SIMV, synchronised intermittent mandatory ventilation.

Overall, 60% of the patients had delayed GE with a mean t50 of 163 ± 7 minutes and GEC of 2.9 ± 0.1. The mean interval from admission to the day of GE measurement was eight days.

### Critical illness factors associated with delayed GE

Table 2 summarises the characteristics of patients who had delayed GE. Slow GE was more common in patients who were older, had higher admission APACHE II scores, admission blood glucose, and bilirubin concentrations, and were ventilated with synchronised intermittent mandatory ventilation (SIMV) mode. On linear regression analysis, GE (both t50 and GEC) correlated with older age, higher admission APACHE II scores, longer length of stay in ICU prior to GE, higher FiO_2 _(fraction of inspired oxygen) requirement, and higher serum creatinine (Table 3). There was no relationship between GE and patient gender, body mass index, ventilatory pressure, APACHE II score on study day, the type of sedation, or requirements for inotropic support.

**Table 2:** Characteristics of patients who had normal or delayed gastric emptying on ^13^C-octanoic acid breath test

	Delayed gastric emptying (*n *= 79)	Normal gastric emptying (*n *= 53)
Age (years)	57.8 ± 2.2^a^	52.2 ± 2.0
Gender (male/female)	58:21	37:16
Body mass index (kg/m^2^)	27.1 ± 0.8	27.9 ± 0.9
Days in ICU prior to study	7.3 ± 0.6	7.4 ± 0.7
APACHE II score		
Admission	24.6 ± 0.6	22.9 ± 0.7
Study day	17.4 ± 0.4	17.8 ± 0.9
Enteral feeding rate (ml/hour)		
Prior to breath testing	44 ± 4^a^	61 ± 5
After breath testing	55 ± 4^a^	60 ± 4
Blood glucose level (mmol/l)		
Admission	9.7 ± 0.9^b^	7.8 ± 0.2
Study day	8.5 ± 0.5	7.7 ± 0.3
Biochemistry		
Albumin (g/l)	23.8 ± 0.6	23.3 ± 0.9
Bilirubin (μmol/l)	24.2 ± 4.0^a^	12.0 ± 1.5
White cell count (× 10^9^/l)	12.3 ± 0.6	13.1 ± 0.7
Serum creatinine (mmol/l)	0.148 ± 0.02	0.113 ± 0.01
Medications, % (*n*)		
Opioid ± benzodiazepine	87% (67)	81% (43)
Propofol	63% (50)	57% (30)
Inotropes	66% (52)	73% (39)
Mechanical ventilation		
SIMV/pressure support (*n*)	49:30^a^	25:28
Fraction of inspired oxygen	0.49 ± 0.02	0.46 ± 0.02
Positive end-expiratory pressure (cm H_2_O)	6.4 ± 0.4	6.8 ± 0.4
Peak inspiratory pressure (cm H_2_O)	24.6 ± 1.0	24.2 ± 1.2
Length of stay (days)		
In ICU	21.0 ± 1.6^b^	13.8 ± 1.2
In hospital	37 ± 2^b^	28 ± 3

^a^*P *< 0.05, versus normal gastric emptying. ^b^*P *< 0.01, versus normal gastric emptying. APACHE II, Acute Physiology and Chronic Health Evaluation II; ICU, intensive care unit; SIMV, synchronised intermittent mandatory ventilation.

**Table 3:** Factors correlated with delayed gastric emptying in critical illness, derived from univariate analyses

	*P *value	*r*
Age	< 0.01	0.32
Admission APACHE II score	< 0.01	0.27
Fraction of inspiratory oxygen	0.02	0.27
Serum creatinine	0.04	0.17
Length of stay in ICU prior to the study	0.02	0.14

APACHE II, Acute Physiology and Chronic Health Evaluation II; ICU, intensive care unit.

### Impact of admission diagnosis on GE

**Figure 1:**
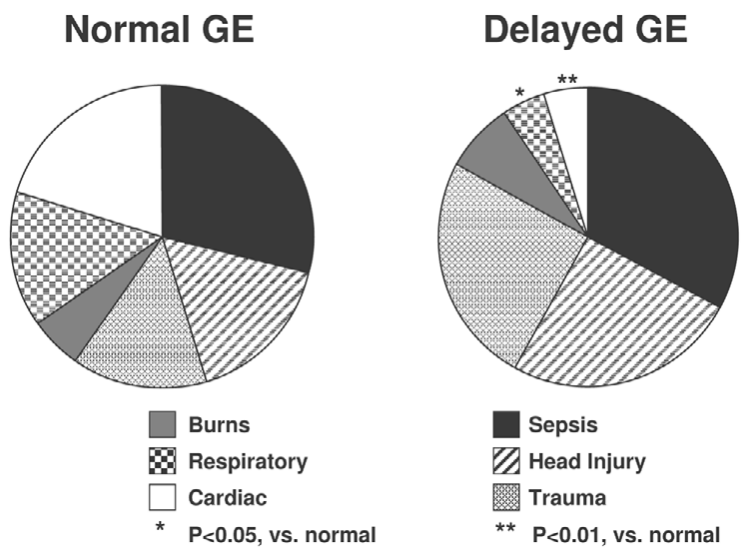
Summary of admission diagnoses in critically ill patients with normal or delayed gastric emptying (GE). *P < 0.05, versus normal GE; **P < 0.01, versus normal GE.

**Figure 2:**
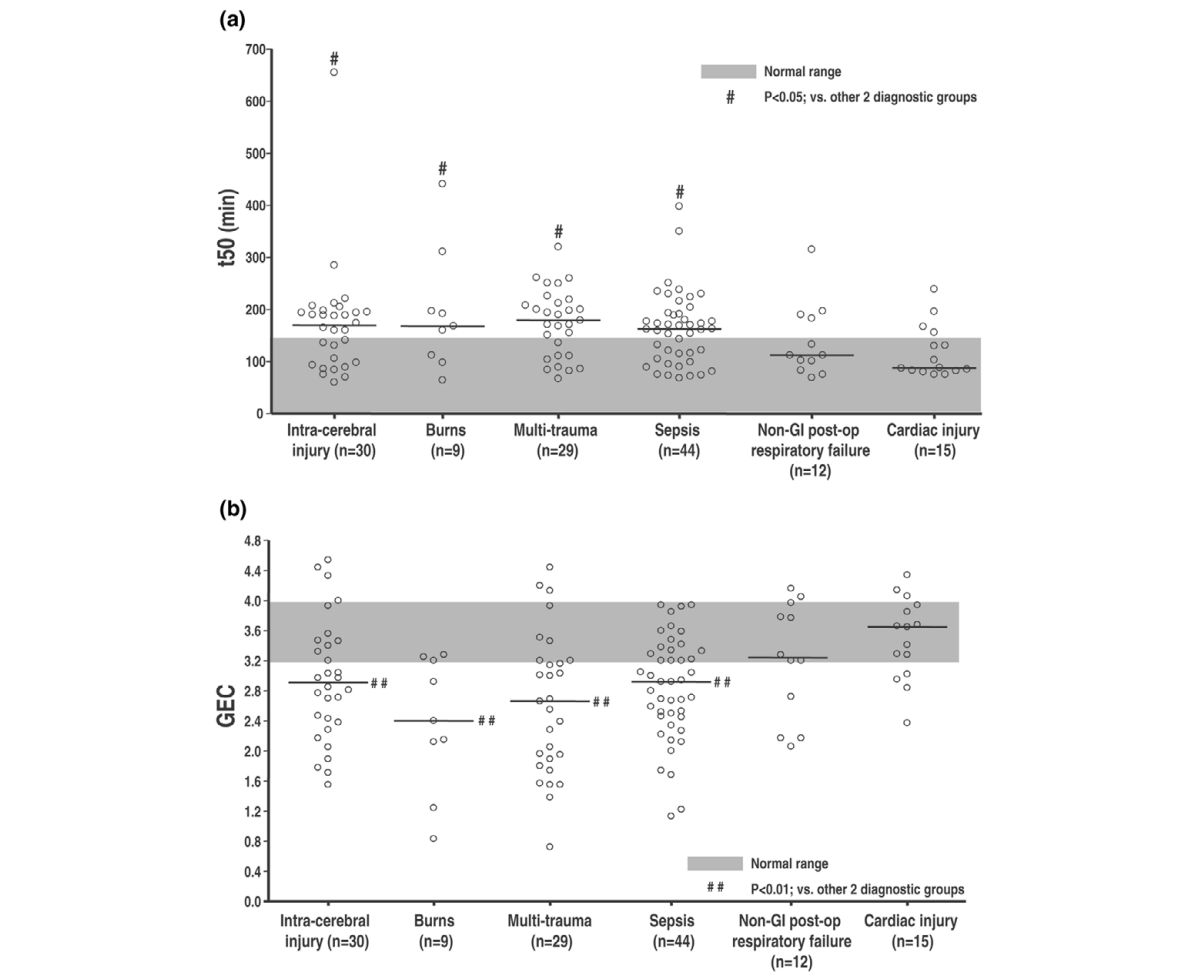
The impact of various admission diagnoses on gastric emptying variables, (a) t50 and (b) GEC, in critically ill patients. The bars represent median values of the gastric emptying variables. ^#^*P *< 0.05, versus cardiac injury and non-gastrointestinal (GI) post-operative respiratory failure; ^##^*P *< 0.01, versus cardiac injury and non-GI post-operative respiratory failure. GEC, gastric emptying coefficient; t50, gastric half-emptying time.

The impact of various admission diagnoses on GE in critical illness is summarised in Table 4 and Figures 1 and 2. After other factors that influence GE were controlled for, there was a modest effect of admission diagnosis on GE (*r *= 0.48; *P *= 0.02) with linear and hierarchical regression analyses. GE was delayed most in patients with burns. Apart from intra-cerebral and burn injuries, delayed GE was also common in patients who were admitted with multi-system trauma and sepsis. In contrast, patients with myocardial injury and non-gastrointestinal post-operative respiratory failure had the lowest incidence of delayed GE.

**Table 4:** Gastric emptying measurements in various groups of diagnosis

	Intra-cerebral injury^a ^ (*n *= 30)	Burns (*n *= 9)	Multi-trauma (*n *= 29)	Sepsis (*n *= 44)	Non-GI post-operative respiratory failure (*n *= 12)	Cardiac injury (*n *= 15)
Delayed gastric emptying	67%	77%	72%	61%	33%^b^	27%^c^
Relative risk of delayed GE^d ^(CI)	1.8 (1.1–2.8)	4.2 (1.1–15.0)	2.0 (1.2–3.5)	1.5 (1.02–2.0)	1.1(0.5–2.8)	
Age (years)	51.5 ± 1.5	37.2 ± 0.9	47.5 ± 1.8	65.8 ± 0.9	59.3 ± 1.2	58.8 ± 1.3
Days in ICU prior to study	9.8 ± 0.6	9.5 ± 1.5	7.0 ± 0.5	7.7 ± 0.5	6.3 ± 0.4	7.5 ± 2.1
APACHE II score						
Admission	23.7 ± 0.4	24.1 ± 0.5	23.2 ± 0.6	25.9 ± 0.5	22.3 ± 0.6	21.9 ± 0.6
Study day	17.5 ± 0.5	14.3 ± 0.6	16.4 ± 0.6	18.5 ± 0.6	17.8 ± 0.5	17.5 ± 0.5
Blood glucose concentrations on study day (mmol/l)	7.9 ± 0.2	8.9 ± 0.3	7.5 ± 0.2	8.5 ± 0.3	7.3 ± 0.4	8.7 ± 0.3
Medications, % (*n*)						
Opioid ± benzodiazepine	80% (24)	89% (8)	89% (26)	82% (36)	83% (10)	73% (11)
Propofol	83% (25)	33% (3)	69% (20)	57% (25)	58% (7)	40% (6)
Inotropes	63% (19)	89% (8)	52% (15)	91% (40)	58% (7)	53% (8)
Enteral feeding rate (ml/hour)						
Prior to breath testing	28 ± 2	47 ± 3	29 ± 3	52 ± 3	53 ± 3	76 ± 3
After breath testing	58 ± 3	67 ± 2	40 ± 2	64 ± 3	55 ± 2	76 ± 2
Length of stay (days)						
In ICU	20 ± 3	34 ± 9	19 ± 2	20 ± 2	20 ± 2	16 ± 3
In hospital	46 ± 6	70 ± 5	52 ± 6	37 ± 3	37 ± 2	29 ± 2
Prokinetic therapy for feeding during ICU admission^e^, % (*n*)	40% (12)	33% (3)	35% (10)	32% (14)	0% (0)	20% (3)

^a^Seven patients had head injury due to multi-trauma. ^b^*P *< 0.05, versus the other four groups on hierarchical regression analysis after controlling for age, admission APACHE II scores, and serum creatinine. ^c^*P *< 0.01, versus the other four groups on hierarchical regression analysis after controlling for age, admission APACHE II scores, and serum creatinine.^d^Compared with patients with cardiac injury.^e^In all patients, prokinetic agents were ceased more than or equal to 24 hours prior to the assessment of GE. APACHE II, Acute Physiology and Chronic Health Evaluation II; CI, 95% confidence interval; GE, gastric emptying; GI, gastrointestinal; ICU, intensive care unit.

### Impact of pre-existing illness on GE

GE was delayed in 58% of patients with no known co-morbidity prior to their ICU admission (t50 = 167 ± 11 minutes and GEC = 2.9 ± 0.1). When age and admission APACHE II scores were controlled for, there was a trend for slow GE to be more common in patients who had pre-existing alcoholic liver disease (80%; *P *= 0.04) and chronic renal failure (75%; *P *= 0.06) and to be less common in patients with known diabetes mellitus (DM) (38%; *P *= 0.05).

### Outcome of delayed GE in critical illness

Patients who had delayed GE received feeds at a lower rate, both before and after the GE assessment (Table 2). The lengths of stay in ICU and in hospital were significantly longer for patients with delayed GE than for those who had normal GE (Table 2). Patients with the most delayed emptying (burns and multi-system trauma) had the longest stays in both ICU and hospital (Table 4). On the other hand, patients with more normal GE (that is, cardiac injury group) had a significantly shorter length of stay in hospital.

## Discussion

The current study is the first to examine the impact of admission diagnosis on GE in critically ill patients by means of the ^13^C-octanoic acid breath test technique for the measurement of GE. Our findings show that admission diagnosis has a significant but modest impact on GE in critically ill patients after controlling for other variables that may influence GE. Consistent with previous reports [2-5,15,17], APACHE II scores, age, length of ICU stay, blood glucose concentrations on admission, renal function, and SIMV mode of mechanical ventilation were associated with delayed GE. Of these, APACHE II scores correlated best with GE, suggesting that illness severity is an important determinant of GE in these patients.

The high prevalence of delayed GE in patients with burns and head injury in the current study is consistent with previous reports [3,4,14-18]. Apart from factors known to slow GE, such as high APACHE II score and the use of opioid and inotropic agents, the precise mechanism underlying the mediation of disturbed emptying in patients with burns and head injuries is unknown. Several neuro-humoral abnormalities related to these injuries, however, may contribute to the gastric dysmotility [27-29]. Thermal injury has been shown to relax the fundus, reduce antral motility, and slow GE due to increases in both sympathetic and opiatergic neural activity and due to release of systemic inflammatory cytokines [2-6,28,29]. In patients with a head injury, raised intra-cranial pressure is thought to be the main mediator of impaired gastric motility and emptying [27].

Previous data relating the impact of sepsis and trauma on GE in critical illness have been limited [30-32]. The observation that GE was delayed in 61% of septic patients has significant clinical implications given that up to 50% of admissions to the ICU are due to sepsis and its complications [2-4]. Early detection and management of feed intolerance in these at-risk patients are important to prevent subsequent complications. In animals, release of inflammatory mediators and cytokines during sepsis has been shown to impair gastric and small intestinal motor activity and is likely to be the main mechanism underlying the impact of sepsis on gut function [33,34].

Our results are also consistent with previous reports on GE in patients admitted following multi-system trauma [31]. The mechanisms underlying the impact of trauma on gastric function are unknown but could also be related to the inhibitory effects that inflammatory mediators and cytokines release during the injuries [32]. This speculation is supported by the observation that GE rates after limb fracture are related to the severity of pain, swelling, and shock associated with the injuries [32]. However, it is also likely that the analgesia requirement in these patients would contribute to the impaired GE. Both opioids and benzodiazepines impair gastric motility and reduce GE [35,36].

In contrast, less than a third of the patients with acute ischaemic myocardial injury had delayed GE. The reason for the low prevalence of delayed GE in this group of patients remains unclear but is unlikely to be related to the lesser degree of illness severity or the lesser use of opioids and inotropes, as these factors were similar to those of other diagnostic groups. In rats, acute myocardial injury delays both GE and intestinal transit of liquids [35]. The hypotension and the release of atrial natriuretic peptide induced by the myocardial infarction are thought to be the main mechanisms that mediate the inhibition of gut motility, possibly via the non-adrenergic and non-cholinergic pathways [37,38].

In the current study, pre-existing co-morbidities had a moderate effect on GE during critical illness. Diseases previously reported to be associated with slow GE, such as liver disease [39] and chronic renal failure [40], increase the likelihood of delayed GE during critical illness. These findings suggest that critical illness may have an 'additive' adverse effect on the disturbed GE in these patients. The exception of DM is intriguing because slow GE is common in patients with both type 1 and 2 DM. However, the lower frequency of delayed GE in our diabetic patients is consistent with a previous report that suggests that GE of liquid is relatively normal in critically ill patients with type 2 DM [41]. The reason for this difference may be related to the different proximal gastric motor responses during critical illness in these patients [42]. In addition, hyperglycaemia is associated with slowing of GE in DM and it is possible that the routinely tight control of blood glucose concentrations may reduce the incidence of delayed GE [43].

The current study has also confirmed that delayed GE in critically ill patients is associated with suboptimal delivery of enteral feeds and longer lengths of stay in ICU and hospital [4,5,7,8]. The adverse effect on successful enteral feeding was most pronounced prior to the assessment of GE (that is, in the first weeks of critical illness), with a mean feeding rate as low as 28 ± 3 ml. Given that the current study is retrospective, the relationship between delayed GE, illness, and its impact on admission outcomes such as lengths of stay in ICU and hospital is unclear, and this requires further study. However, feed intolerance, an indirect marker of delayed GE, has been associated with prolonged ICU stay, which may be related to the increased risk of gastro-oesophageal reflux and subsequent aspiration pneumonia [3,4,10].

There are some limitations to the current study. Due to the retrospective nature of the study, selection bias cannot be totally excluded. However, such bias is likely to be minimal given that the studies into which patients were recruited had similar inclusion and exclusion criteria and that data for more than 90% of the patients were available for analysis. GE was assessed only once, approximately 1 week after admission. The variation in severity of illness and prescribed medications throughout the ICU course may lead to fluctuation in GE in critically ill patients over time. The relatively wide time window between admission and measurement of GE could have potentially weakened the strength of association between admission diagnosis and GE. However, because the association remained at day eight with other factors known to alter GE in critical illness having been controlled for, the relationship between admission diagnosis and GE may have been even stronger if GE had been assessed within the first three days of admission. Currently, there are no data on the temporal relationship between the impact of admission diagnosis and GE in critically ill patients. Finally, the use of the ^13^C-octanoic acid breath test to measure GE is also a potential source of error. However, a number of studies, in both healthy humans and critically ill patients, have demonstrated a strong correlation between this technique and the current gold standard technique, gastric scintigraphy [19,20,22].

## Conclusion

Admission diagnosis has a modest impact on GE in critically ill patients, even after controlling for factors such as age, illness severity, and medication, which are known to influence this function. Apart from burns and head injury, patients with sepsis and multi-system trauma are also at high risk of delayed GE. Patients with these admission diagnoses should be monitored carefully for signs of feed intolerance during enteral feeding so that treatment for feed intolerance can be instituted early to prevent reflux complications and optimise nutritional support.

## Key messages

• Delayed GE is common in critically ill patients (60%) and is associated with suboptimal delivery of enteral feeds and longer lengths of stay in ICU and hospital.

• Admission diagnosis has a modest impact on GE in critically ill patients, even after controlling for factors that are known to influence this function: age, illness severity, and medication.

• Apart from burns and head injury, patients with sepsis and multi-system trauma are also at high risk of delayed GE.

• Patients with 'high-risk' admission diagnoses should be monitored carefully for signs of feed intolerance during enteral feeding so that treatment for feed intolerance can be instituted early to prevent reflux complications and optimise nutritional support.

## Abbreviations

APACHE II = Acute Physiology and Chronic Health Evaluation II; DM = diabetes mellitus; GE = gastric emptying; GEC = gastric emptying coefficient; ICU = intensive care unit; SIMV = synchronised intermittent mandatory ventilation; SIRS = systemic inflammatory response syndrome; t50 = gastric half-emptying time.

## Competing interests

The authors declare that they have no competing interests.

## Authors' contributions

NQN was the main contributor to the conception and design of the study, the analysis and interpretation of the data, and the drafting of the manuscript. MPN contributed to the acquisition and analysis of the data. MC, RJF, and RHH contributed to the conception and design of the study and the revision of the manuscript. All authors read and approved the final manuscript.
